# Utilization of Digital PCR in Quantity Verification of Plasmid Standards Used in Quantitative PCR

**DOI:** 10.3389/fmolb.2020.00155

**Published:** 2020-07-29

**Authors:** Martina Beinhauerova, Vladimir Babak, Barbara Bertasi, Maria Beatrice Boniotti, Petr Kralik

**Affiliations:** ^1^Department of Food and Feed Safety, Veterinary Research Institute, Brno, Czechia; ^2^Department of Experimental Biology, Faculty of Science, Masaryk University, Brno, Czechia; ^3^Controllo Alimenti, Istituto Zooprofilattico Sperimentale della Lombardia e dell'Emilia Romagna, Brescia, Italy; ^4^Tecnologie Biologiche Applicate, Istituto Zooprofilattico Sperimentale della Lombardia e dell'Emilia Romagna, Brescia, Italy; ^5^Department of Hygiene and Technology of Food of Animal Origin and of Gastronomy, Faculty of Veterinary Hygiene and Ecology, University of Veterinary and Pharmaceutical Sciences, Brno, Czechia

**Keywords:** digital PCR, absolute quantification, quantity verification, quantification plasmid standard, qPCR, real time PCR

## Abstract

Quantitative PCR (qPCR) is a widely used method for nucleic acid quantification of various pathogenic microorganisms. For absolute quantification of microbial load by qPCR, it is essential to create a calibration curve from accurately quantified quantification standards, from which the number of pathogens in a sample is derived. Spectrophotometric measurement of absorbance is a routine method for estimating nucleic acid concentration, however, it may be affected by presence of other potentially contaminating nucleic acids or proteins and salts. Therefore, absorbance measurement is not reliable for estimating the concentration of stock solutions of quantification standards, based on which they are subsequently diluted. In this study, we utilized digital PCR (dPCR) for absolute quantification of qPCR plasmid standards and thus detecting possible discrepancies in the determination of the plasmid DNA number of standards derived from UV spectrophotometry. The concept of dPCR utilization for quantification of standards was applied on 45 qPCR assays using droplet-based and chip-based dPCR platforms. Using dPCR, we found that spectrophotometry overestimated the concentrations of standard stock solutions in the majority of cases. Furthermore, batch-to-batch variation in standard quantity was revealed, as well as quantitative changes in standards over time. Finally, it was demonstrated that droplet-based dPCR is a suitable tool for achieving defined quantity of quantification plasmid standards and ensuring the quantity over time, which is crucial for acquiring homogenous, reproducible and comparable quantitative data by qPCR.

## Introduction

Quantitative PCR (qPCR) is currently the method of choice for nucleic acid detection and quantification of various microbial pathogens due to its high sensitivity, specificity, reproducibility, and wide dynamic range. Its theoretical limit of detection was set at three copies per qPCR reaction, assuming its ability to detect a single copy of the target nucleic acid (Bustin et al., 2009). qPCR is used in various applications in a wide range of areas including food safety and healthcare (Kralik et al., 2011; Alidjinou et al., 2017; Walker et al., 2017). In order to determine the absolute number of microbial pathogens in a sample using qPCR, it is necessary to create a calibration curve derived from a serially diluted quantification standard containing a known amount of copies or concentration of plasmids, genomic DNA or other nucleic acid molecules carrying target genes. Therefore, the exact assessment of the amount of nucleic acid copies or concentration of standards is essential for the correct quantification of pathogens in a sample. Typically, the concentration of nucleic acid of a standard is determined based on spectrophotometric or fluorometric measurements of nucleic acid absorbance or fluorescence, respectively (Kline et al., 2009), subsequently the number of DNA copies can be calculated according to the molecular weight of DNA material. However, low purity may influence the results of absorbance measurements. The concentration of nucleic acid measured may be altered by residues of DNA or RNA, proteins, and salts, which may lead to production of the standards containing incorrect amount of nucleic acid copies (Sanders et al., 2011). Currently, there is no standardized protocol for independent quantity verification of desired DNA in qPCR standards and ensuring measurement accuracy in qPCR. Moreover, since quantification standards are not uniform among laboratories performing quantification of pathogenic microorganisms using qPCR, the determination of the number of pathogens in various laboratories using different qPCR standards is not comparable, which is then reflected in differing interpretations of results (Pavsic et al., 2015).

In addition to accurately quantified qPCR standards, a number of other factors that may affect the qPCR quantification of target genes cannot be omitted. The choice of nucleic acid isolation method has a distinctive effect on subsequent qPCR quantification of pathogens in sample and thus the use of different methods may lead to gene number variability (Smith et al., 2006). Furthermore, possibility of additional variability arising from the diverse reagents and instruments used and different calibration curves should be taken into account in absolute qPCR quantification (Bustin et al., 2009). Therefore, direct comparison of the absolute gene copy numbers determined in different qPCR assays and different standard curves should be made with caution (Smith et al., 2006). Besides, another disadvantage of qPCR-based methods is the need for sequence information about a specific target gene enabling the design of primers and probes, therefore it can only be applied on already known genes (Smith and Osborn, 2009).

Digital PCR (dPCR) nowadays represents one of the most powerful tools for absolute nucleic acid quantification, which does not require the creation of a standard curve (Hindson et al., 2011). dPCR has become a widely used method that offers a number of advantages for detection and quantification of nucleic acids (Gerdes et al., 2016). It is used for molecular analyses in clinical as well as research applications, such as for the detection of microRNAs associated with cancer (Ma et al., 2013), chromosomal abnormalities (Zimmermann et al., 2008), quantification of pathogenic bacteria (Porcellato et al., 2016; Talarico et al., 2016), viral load (Lui and Tan, 2014; Nicot et al., 2016), testing of genetically modified organisms (Morisset et al., 2013), and next-generation sequencing (White et al., 2009). The principle of dPCR utilizes a limiting dilution and random sample distribution into hundreds to millions of uniformly sized nanoliter or picoliter separate reaction partitions, in which the target nucleic acid sequence is amplified. Currently, there are several available dPCR platforms that differ mainly in the arrangement of reaction partitions. The reaction partitions may be either microfluidic chambers or microwells placed on a microchip (chip-based digital PCR, cdPCR) or water-in-oil emulsion droplets (droplet-based digital PCR, ddPCR). Quantification of target nucleic acid sequence is based on counting the number of positive (sequence detected) and negative (sequence not detected) reaction partitions after previous amplification with the correction to real numbers utilizing Poisson distribution (Hindson et al., 2011). The advantages of dPCR are high sensitivity and precision, tolerance to inhibitors (Huggett et al., 2013; Lui and Tan, 2014) and increased signal-to-noise ratio due to partitioning of the sample, thereby the background signal is diluted out and it is thus possible to detect low-abundance targets (Sanders et al., 2011).

The main aim of this study was to evaluate the suitability of dPCR utilization for independent quantity verification of quantification plasmid standards used in qPCR, more specifically, for absolute quantification of the standards and thus the estimation of discrepancies in the determination of the plasmid DNA number of standards derived from spectrophotometric absorbance measurements. The concept of dPCR as a promising technique for accurate quantification of standards was applied on a panel of 45 qPCR assays, comparing the performance of ddPCR and cdPCR platforms. Batch-to-batch variation in standard quantity was investigated, as well as possible quantitative changes in standards over time when stored at −20°C. Furthermore, the effect of conformation structure of the quantification plasmid standards (circular and linear form of plasmid DNA) on the quantity estimation by dPCR and differences with qPCR amplification was examined. Several plasmid isolation kits commercially available were tested for their ability to remove possible contamination affecting absorbance measurement of plasmid standard stock solutions. Finally, this study demonstrated that ddPCR could be a suitable tool for achieving defined quantity of quantification plasmid standards and ensuring the quantity over time, which is essential to obtain homogenous, reproducible, and comparable quantitative data by qPCR in various commercial and research laboratories.

## Materials and Methods

### Preparation of Quantification Standards

Quantification plasmid standards of 45 qPCR assays utilized for the detection and quantification of various bacterial, viral, and parasitical agents (Table 1 and Supplementary Table 1) were prepared by the cloning of a specific nucleotide sequence of a particular microbial pathogen to pDRIVE plasmid vector and transformed to chemocompetent *Escherichia coli* (*E. coli*) TOP10 cells (both supplied by Qiagen, Germany). Clones carrying specific plasmids were propagated 16 h in Luria-Bertani broth (Sigma-Aldrich, USA) containing 50 μg/ml of kanamycin (Sigma-Aldrich) at 37°C with shaking. The culture was centrifuged at 6,800 × g for 3 min at room temperature and plasmid DNA was isolated from the prepared pellet using QIAprep Spin Miniprep Kit (Qiagen) according to the manufacturer's instructions.

**Table 1:** The list of quantification standards of 45 qPCR assays utilized in this study.

**Microorganism**	**Target locus**	**Abbreviation**
*Bacillus anthracis*	BA5357	BA5357
	pagA	BA pag
*Brucella* spp.	BCSP31	BCSP31
	omp2	B omp
*Campylobacter coli*	glyA	Camp col
*Campylobacter jejuni*	hipO	Camp jej
*Campylobacter lari*	bipA	Camp lar
*Campylobacter upsaliensis*	bipA	Camp ups
*Clostridium botulinum*	16S rDNA	CB 16S
*Clostridium difficile*	Tpi	CD tpi
*Clostridium perfringens*	Cpa	CP cpa
*Clostridium* spp.	16S rDNA	CP 16S
*Clostridium tetani*	Tetox	Clos tet
*Cronobacter sakazakii*	rpsU gene 3′ end and the primase (dnaG) gene 5′ end	Crono rps
	rpoB	Crono rpo
*Cryptosporidium* spp.	hsp70	Cryp par
*Erysipelothrix rhusiopathiae*	ERH 1059	ERH 1059
	Soda	ERH sod
*Escherichia coli*	uidA	EC uid
	rfbE	EC rfbe
*Giardia lamblia*	β-giardin	GL
Human adenovirus	Hexon	AdV hex
Human adenovirus (serotype 40 and 41)	Fiber	AdV fib
*Listeria monocytogenes*	hlyIII	LM hly
*Listeria* spp.	23S rDNA	LM 23S
*Mycobacterium avium* complex	IS1311	MAC IS1311
*Mycobacterium avium* ssp. *avium*	IS901	MAA IS901
*Mycobacterium avium* ssp. *hominissuis* and *Mycobacterium avium* ssp. *avium*	IS1245	MAHA IS1245
*Mycobacterium avium* ssp. *paratuberculosis*	IS900	MAP IS900
	F57	MAP F57
*Mycobacterium* spp.	ITS	Myco ITS
*Mycobacterium tuberculosis*	devR	Myco dev
*Pseudomonas aeruginosa*	gyrB	PA gyr
	ecfX	PA ecf
*Pseudorabies virus*	gB	PRV
*Salmonella enterica*	Ttr	SE ttr
*Staphylococcus aureus*	SA442	SA442
	Nuc	SA nuc
*Toxoplasma gondii*	B1 gene	T gon
Verotoxigenic *Escherichia coli*	stx 1, 2	VTEC stx
	Eae	VTEC eae
*Yersinia enterocolitica*	Ail	YE ail
*Yersinia pestis*	caf1	YP caf
	Pla	YP pla
*Yersinia* spp.	ompF	YE omp

Isolated plasmids were divided into two aliquots and one was linearized using *Bam*HI restriction endonuclease (New England Biolabs, USA). The absence of the *Bam*HI restriction enzyme site within all the plasmid inserts was checked by the Webcutter online tool (http://www.firstmarket.com/cutter/cut2.html) prior to experimental work. The restriction enzyme digest reaction was composed of 10 μg plasmid DNA, 5 μl NEBuffer 3.1 (New England Biolabs), and 100 U *Bam*HI in a final volume of 50 μl. The enzymatic reaction was carried out for 2 h at 37°C, subsequently, the linearized plasmid DNA was purified using QIAquick PCR Purification Kit (Qiagen) according to the manufacturer's instructions. Linearization was verified by agarose gel electrophoresis (1%) and staining with ethidium bromide. The second non-linearized aliquot of plasmids underwent the same procedure of linearization and purification with the only exception that the restriction enzyme was replaced with an identical volume of water in the digestion reaction.

The concentration of the purified plasmid DNA was determined by spectrophotometric measurement of nucleic acid absorbance using NanoDrop 2000c (Thermo Scientific, USA). Then both linearized and non-linearized plasmid DNA stock solutions were ten-fold serially diluted in Tris-EDTA buffer (Amresco, USA) with Carrier DNA solution (salmon sperm DNA, 50 ng/μl; Serva, Germany) to a gradient of the standards with expected concentrations in a range of 10^5^–10^0^ copies/μl and stored at −20°C. This was followed by quantification of diluted plasmid standards using qPCR and dPCR assays.

To investigate a possible effect of the plasmid purification kit on the quality of the isolated plasmids in terms of presence of contaminating DNA affecting absorbance measurement of stock solution and subsequent dilution of the standards, six different kits for the plasmid DNA purification commercially available—QIAprep Spin Miniprep Kit already mentioned above, NucleoSpin Plasmid (Macherey-Nagel, Germany), Monarch Plasmid Miniprep Kit (New England BioLabs, USA), GeneJET Plasmid Miniprep Kit (Thermo Scientific, USA), High Pure Plasmid Isolation Kit (Sigma-Aldrich, USA), and GenElute Plasmid Miniprep Kit (Sigma-Aldrich, USA)—were compared on the standards used for detection of human adenovirus (AdV fib) and *Staphylococcus aureus* (SA442), selected from a list of pathogens (Table 1). A single batch of the propagated *E. coli* cells with the inserted plasmids was split into six aliquots and each underwent plasmid purification by the respective kit according to the manufacturer's recommendations. The subsequent procedure was the same as mentioned above, isolated plasmid standards were linearized, then purified by QIAquick PCR Purification Kit, diluted based on spectrophotometric estimation to concentrations 10^4^, 10^3^, and 10^2^ copies/μl and quantified using ddPCR assay.

### Comparison of the Performance of the Circular and Linear Forms of Plasmids in qPCR Assays

To evaluate whether there is a difference in amplification of linear and circular plasmid standards, four plasmid standards containing specific sequence from pathogen (Table 1) were selected—*Campylobacter jejuni* (Camp jej), *Campylobacter lari* (Camp lar), human adenovirus (AdV fib), and *S. aureus* (SA442). The quantity of the circular and linear form of these four plasmid standards, which were ten-fold serially diluted based on spectrophotometric estimation in a range of 10^5^–10^0^ copies/μl was determined using qPCR. The experiment was run in independent biological duplicates comprising the whole procedure of transformed cells propagation, plasmid purification, and spectrophotometric determination (two batches of each of the four standards differing in preparation time). The linear plasmids quantified using ddPCR were utilized for construction of standard curves in qPCR.

qPCR reaction mixtures used in this study consisted of 10 μl of LightCycler 480 Probes Master (Roche, Czech Republic), 1 U of Uracil DNA Glycosylase (Roche), 50 nM of TaqMan probe, 500 nM of each of primers, and 5 μl of plasmid DNA in a total volume of 20 μl. The qPCR assays were run in duplicate for each analyzed sample (each dilution) using the LightCycler 480 instrument (Roche) under the following reaction conditions: initial denaturation at 95°C for 7 min, followed by 45 amplification cycles at 95°C for 10 s, 60°C for 30 s, and 72°C for 1 s. The subsequent analysis of the results was performed using the “Fit point analysis” option of the LightCycler 480 Software (1.5.0.39).

### Comparison of the Performance of the Circular and Linear Forms of Plasmids in ddPCR Assays

Similarly to qPCR, linear and circular forms of plasmid standards (again two batches of each standard) were investigated on the four model assays. The manner of the testing was identical to qPCR, but the range of concentrations tested by ddPCR was 10^4^, 10^3^, and 10^2^ copies/μl as expected based on spectrophotometric estimation.

ddPCR was carried out using the QX200 droplet digital PCR system (Bio-Rad, USA) according to the manufacturer's instructions. The assays were run in duplicate for each analyzed sample (each dilution) in a total volume of 22 μl. The reaction mix contained 11 μl of ddPCR Supermix for Probes (No UTP), 5 μl of plasmid DNA and the same probes and primers at the same concentrations, 50 and 500 nM, respectively, as in qPCR. The reaction mix and 70 μl of droplet generation oil were loaded into wells of the DG8 cartridge and placed into the QX200 Droplet generator. Forty microliters of generated droplets were transferred into 96-well PCR plate, which was then sealed using foil heat seal in PX1 PCR plate sealer (Bio-Rad, USA) and placed in a T100 Thermal Cycler (Bio-Rad, USA) for PCR. Cycling conditions were enzyme activation at 95°C for 10 min, followed by 40 cycles of a two step thermal profile at 94°C for 30 s and 60°C for 1 min and a final enzyme inactivation at 98°C for 10 min while maintaining ramp rate 2°C/s. After thermal cycling, the 96-well plate was placed in the QX200 Droplet Reader, where droplets were read and analyzed using QuantaSoft Software (1.7.4.0917). From the values measured by ddPCR, a maximum likelihood estimate (MLE) of the plasmid concentration was calculated according to Equation (1) and plotted against the theoretical quantity according to Equation (2) as mentioned below (Statistical and Mathematical Analysis).

### Comparison of the ddPCR and cdPCR Plasmid Quantification on 45 qPCR Assays Intended for the Detection and Quantification of Various Viral, Bacterial, and Parasitical Agents

Both dPCR platforms, ddPCR and cdPCR, were used for the quantification of plasmid standards diluted based on spectrophotometric estimation of concentrations at 10^4^, 10^3^, and 10^2^ copies/μl. ddPCR assays were performed as stated above.

cdPCR was performed with the QuantStudio 3D Digital PCR System (Applied Biosystems, USA). cdPCR assays were run in duplicate for each dilution. The reaction mix at a total volume of 16 μl was composed of 8 μl of QuantStudio 3D Digital PCR Master Mix v2, 5 μl of plasmid DNA and again the same probes and primers at the same concentrations as in qPCR and ddPCR. Then, 14.5 μl of the reaction mixture was loaded on a QuantStudio 3D Digital PCR Chip v2 using QuantStudio 3D Digital PCR Chip Loader. The chips were placed in ProFlex 2x Flat PCR System for thermal cycling under the conditions: 96°C for 10 min, followed by 39 cycles of a two step thermal profile at 60°C for 2 min, and 98°C for 30 s and the final extension step at 60°C for 2 min. Thereafter, the chips were read with the QuantStudio 3D Digital PCR Instrument and analyzed with the QuantStudio 3D AnalysisSuite Software web application. As with ddPCR, MLE of concentration was calculated and plotted against the theoretical quantity according to Equations (1) and (2), respectively.

### Comparison of the Concentrations of 5 Selected Linear Plasmid Standards Diluted Based on Spectrophotometric Estimation and Re-diluted Based on Value (MLE) Determined by ddPCR

After quantification of 45 linear plasmid standards diluted based on spectrophotometric estimation using dPCR, five standards were selected—AdV fib, Camp lar, Crono rps, EC uid, and MAP F57—to be re-diluted based on the MLE of the concentration calculated from values measured by ddPCR (Equation 1). After re-dilution, the concentrations of plasmid standards were again measured by ddPCR and plotted against the theoretical quantity (Equation 2).

### Evaluation of Stability of Linear Plasmid Standards During Long-Term Storage

To evaluate whether the quantity of linear plasmid standards changes during long-term storage, stock solutions of purified linearized plasmids from the initial experiment with four qPCR systems comparing circular and linear plasmid form were reassessed after 19 (first batch) or 14 (second batch) months of storage at −20°C. Each solution was again diluted based on previously determined spectrophotometric estimation and copy number of the plasmids was determined by ddPCR. Subsequently, calculated MLE (Equation 1) of the plasmid concentration from the values measured by the second ddPCR assay was considered a reference value and served for the preparation of the newly diluted plasmid standards, which were again measured by ddPCR and MLE calculated. The values obtained were plotted against the theoretical quantity (Equation 2).

### Statistical and Mathematical Analysis

#### Calculation of MLE of the Plasmid Concentration

The measured concentrations of ten-fold serially diluted standards should follow Poisson's distribution. Assuming that the concentration of the plasmid standard in the initial sample is equal to λ, then the sample on the i-th ten-fold dilution (i = 1, 2, 3, …) has a Poisson's distribution Po (λ/10^i^) with an expected value λ/10^i^. For example, the measured values *x, y*, and *z* corresponding to ten-fold serial dilution four, five, and six, respectively, should be approximately equal to the expected values λ/10^4^, λ/10^5^, and λ/10^6^, from which three estimates of the initial concentration λ can be made: *x* × 10^4^, *y* × 10^5^, and *z* × 10^6^. The maximum likelihood estimate (MLE) of concentration λ is the arithmetic average of the individual estimations, which can be calculated as follows:

(1) $$\begin{array}{r} \lambda =\left(x\times 10^{4}+y\times 10^{5}+z\times 10^{6}\right)/3 \end{array}$$

As the value *y* corresponds to a dilution five, it should be recalculated to λ/10^5^.

#### Comparison of the Concentration Estimates With the Required Concentration of Plasmid Standards

MLE of the concentration calculated from the values measured by dPCR according to Equation (1) was compared to the theoretical quantity according to this quotient:

(2) $$x\%=\frac{\text{MLE of the concentration}}{\text{theoretical quantity}}\;\times 100$$

The theoretical quantity represents the individual concentrations of the standard dilution series expected to be obtained after dilution of the standard stock solution, i.e., ten-fold serial dilutions in the range of 10^5^–10^0^ copies/μl.

Statistical analysis of ddPCR and cdPCR was performed by a statistical software GraphPad Prism 5.04 (GraphPad Software, Inc., San Diego, CA, USA). *P*-value differences lower than 0.05 were considered statistically significant.

## Results

### Quantification of Circular and Linear Plasmid Standards Using ddPCR

**Figure 1:**
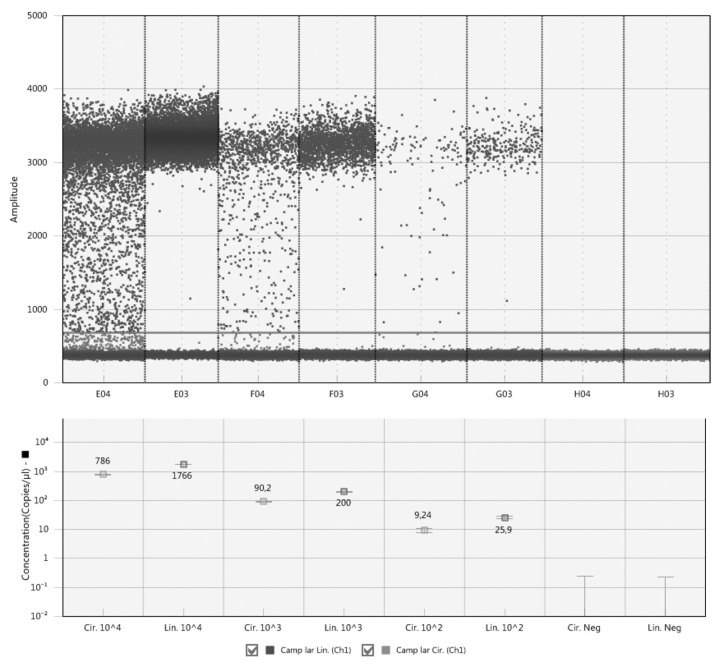
Comparison of circular and linear form of plasmid standards by ddPCR. The circular plasmids are show in columns E04, F04, and G04, the linear ones in columns E03, F03, and G03. H04 and H03 are corresponding negative controls. The line across all columns indicates a threshold line separating positive and negative droplets situated above and below the line, respectively.

Comparison of the circular and linear plasmid standards using ddPCR failed. The concentration of circular plasmid standards could not be accurately determined by ddPCR due to the so-called rain effect, which is characterized by droplets exhibiting fluorescence ranging between those of explicit negative and positive droplets, which makes it difficult to set the threshold correctly (Figure 1). In order to set the threshold correctly, the plasmid standards need to be linearized, which corresponds with the manufacturer's recommendation, and therefore further analyzes using dPCR were performed only with linearized plasmid standards.

MLE of the linear plasmid concentration was calculated according to Equation (1), and then compared to the theoretical quantity according to Equation (2). E.g., for Camp jej, calculation of MLE of plasmid concentration based on three estimates of the initial concentration λ is as follows: λ_MLE_ = (5,080 × 10^4^ + 555 × 10^5^ + 64.6 × 10^6^)/3 = 5.697 × 10^7^ (Table 2). As for the value 555, which corresponds to a dilution five, it should be recalculated to λ/10^5^, i.e., 5.697 × 10^7^/10^5^ = 569.7. Similarly, values for dilutions four and six were calculated. By percentage comparison of MLE of the concentration with the theoretical quantity, a value of 57% was obtained.

**Table 2:** The quantity value of the linearized plasmid standards diluted based on spectrophotometric estimation determined by ddPCR.

**Standard**	**Theoretical quantity (copies/μL)**	**1st batch**			**2nd batch**		
		**Measured conc. (copies/μL)**	**MLE of conc. (copies/μL)^a^**	**Ratio (%)^b^**	**Measured conc. (copies/μL)**	**MLE of conc. (copies/μL)^a^**	**Ratio (%)^b^**
Camp jej	10^4^	5.08 × 10^3^	5.70 × 10^3^	57	6.93 × 10^3^	7.39 × 10^3^	74
	10^3^	5.55 × 10^2^	5.70 × 10^2^		7.20 × 10^2^	7.39 × 10^2^	
	10^2^	6.46 × 10^1^	5.70 × 10^1^		8.04 × 10^1^	7.39 × 10^1^	
Camp lar	10^4^	7.78 × 10^3^	9.14 × 10^3^	91	5.06 × 10^3^	5.30 × 10^3^	53
	10^3^	8.64 × 10^2^	9.14 × 10^2^		5.35 × 10^2^	5.30 × 10^2^	
	10^2^	1.10 × 10^2^	9.14 × 10^1^		5.49 × 10^1^	5.30 × 10^1^	
AdV fib	10^4^	8.35 × 10^3^	9.00 × 10^3^	90	5.16 × 10^3^	5.12 × 10^3^	51
	10^3^	9.29 × 10^2^	9.00 × 10^2^		5.10 × 10^2^	5.12 × 10^2^	
	10^2^	9.36 × 10^1^	9.00 × 10^1^		5.12 × 10^1^	5.12 × 10^1^	
SA442	10^4^	6.58 × 10^3^	7.13 × 10^3^	71	5.10 × 10^3^	5.48 × 10^3^	55
	10^3^	7.07 × 10^2^	7.13 × 10^2^		5.57 × 10^2^	5.48 × 10^2^	
	10^2^	7.74 × 10^1^	7.13 × 10^1^		5.77 × 10^1^	5.48 × 10^1^	

a *MLE (maximum likelihood estimate) of concentration was calculated as arithmetic average of the measured concentration values of the three standard dilutions converted to the same order*.

b *The ratio expresses the percentage of MLE of plasmid standard concentration to the theoretical quantity*.

Using ddPCR, it was found that the concentrations of four selected linear plasmid standards—Camp jej, Camp lar, AdV fib, and SA442—diluted based on spectrophotometric estimation corresponded to a theoretical quantity of 57, 91, 90, and 71%, respectively, in the first batch, and of 74, 53, 51, and 55%, respectively, in the second batch (Table 2).

### Comparison of Circular and Linear Plasmid Standards by qPCR

The concentration of the circular form of the plasmid standards determined by qPCR was lower compared to the linear form in all cases (Table 3). The percentage of circular plasmid concentration to linear plasmid concentration was in the range of about 20–30%.

**Table 3:** Comparison of quantity value of circular and linear form of the plasmid standards by qPCR.

**Standard**	**Theoretical quantity (copies/μL)**	**1st batch**				**2nd batch**			
		**Linear** **(copies/μL)**	**Circular (copies/μL)**	**Ratio (%)^a^**	**Mean** **ratio (%)**	**Linear (copies/μL)**	**Circular (copies/μL)**	**Ratio (%)^a^**	**Mean** **ratio (%)**
Camp jej	10^5^	5.29 × 10^4^	1.21 × 10^4^	23	24	6.69 × 10^4^	1.22 × 10^4^	18	18
	10^4^	4.60 × 10^3^	1.05 × 10^3^	23		7.04 × 10^3^	1.24 × 10^3^	18	
	10^3^	4.70 × 10^2^	1.09 × 10^2^	23		7.11 × 10^2^	1.17 × 10^2^	16	
	10^2^	5.55 × 10^1^	1.14 × 10^1^	21		7.48 × 10^1^	1.36 × 10^1^	18	
	10^1^	5.38 × 10^0^	1.28 × 10^0^	24		7.33 × 10^0^	1.27 × 10^0^	17	
	10^0^	5.01 × 10^−1^	1.61 × 10^−1^	32		6.51 × 10^−1^	-	-	
Camp lar	10^5^	8.23 × 10^4^	1.69 × 10^4^	21	25	5.25 × 10^4^	9.26 × 10^3^	18	16
	10^4^	8.62 × 10^3^	2.00 × 10^3^	23		4.60 × 10^3^	7.31 × 10^2^	16	
	10^3^	8.20 × 10^2^	2.10 × 10^2^	26		5.32 × 10^2^	6.77 × 10^1^	13	
	10^2^	6.84 × 10^1^	2.20 × 10^1^	32		5.03 × 10^1^	7.57 × 10^0^	15	
	10^1^	7.06 × 10^0^	2.68 × 10^0^	38		7.04 × 10^0^	1.36 × 10^0^	19	
	10^0^	9.83 × 10^−1^	1.30 × 10^−1^	13		4.17 × 10^−1^	-	-	
AdV fib	10^5^	8.71 × 10^4^	1.80 × 10^4^	21	26	5.49 × 10^4^	1.36 × 10^4^	25	24
	10^4^	8.12 × 10^3^	1.79 × 10^3^	22		5.39 × 10^3^	1.39 × 10^3^	24	
	10^3^	9.04 × 10^2^	1.48 × 10^2^	16		4.18 × 10^2^	1.14 × 10^2^	27	
	10^2^	8.77 × 10^1^	1.86 × 10^1^	21		5.11 × 10^1^	1.12 × 10^1^	22	
	10^1^	8.46 × 10^0^	1.24 × 10^0^	15		5.84 × 10^0^	1.39 × 10^0^	24	
	10^0^	9.80 × 10^−1^	6.16 × 10^−1^	63		5.19 × 10^−1^	-	-	
SA442	10^5^	6.95 × 10^4^	1.21 × 10^4^	17	19	5.20 × 10^4^	1.15 × 10^4^	22	20
	10^4^	6.73 × 10^3^	1.22 × 10^3^	18		5.06 × 10^3^	9.18 × 10^2^	18	
	10^3^	6.18 × 10^2^	1.16 × 10^2^	19		5.38 × 10^2^	8.79 × 10^1^	16	
	10^2^	6.76 × 10^1^	9.93 × 10^0^	15		5.40 × 10^1^	9.77 × 10^0^	18	
	10^1^	6.99 × 10^0^	1.35 × 10^0^	19		5.04 × 10^0^	1.22 × 10^0^	24	
	10^0^	6.68 × 10^−1^	1.91 × 10^−1^	29		7.56 × 10^−1^	1.73 × 10^−1^	23	

*The quantity value of the linear plasmids was determined by ddPCR and these linear plasmids served as the standards for qPCR quantification*.

a *The ratio expresses the percentage of circular plasmid concentration to linear plasmid concentration of the standards*.

### Comparison of Six Different Kits for Plasmid DNA Isolation by ddPCR

**Figure 2:**
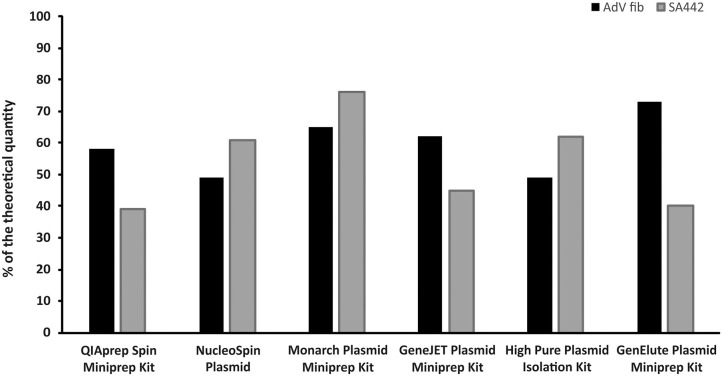
Comparison of six different kits for plasmid DNA isolation. The bars represent the calculated MLE of plasmid standard concentration compared to the theoretical concentration (the values are given in percent).

Differences were found in the concentrations of linear plasmid standards diluted based on spectrophotometric estimation after prior isolation by six different isolation kits, as well as between two standards isolated by the same kit (Figure 2). The concentrations of linear plasmid standards differed from a theoretical quantity in the range of 24–61% and individual plasmid standards when using various kits differed from each other by up to approximately 50%. The removal of contamination affecting the absorbance measurements of standard stock solutions was apparently not achieved using any of the kits.

### Quantification of 45 Linear Plasmid Standards Using ddPCR and cdPCR Assays

**Figure 3:**
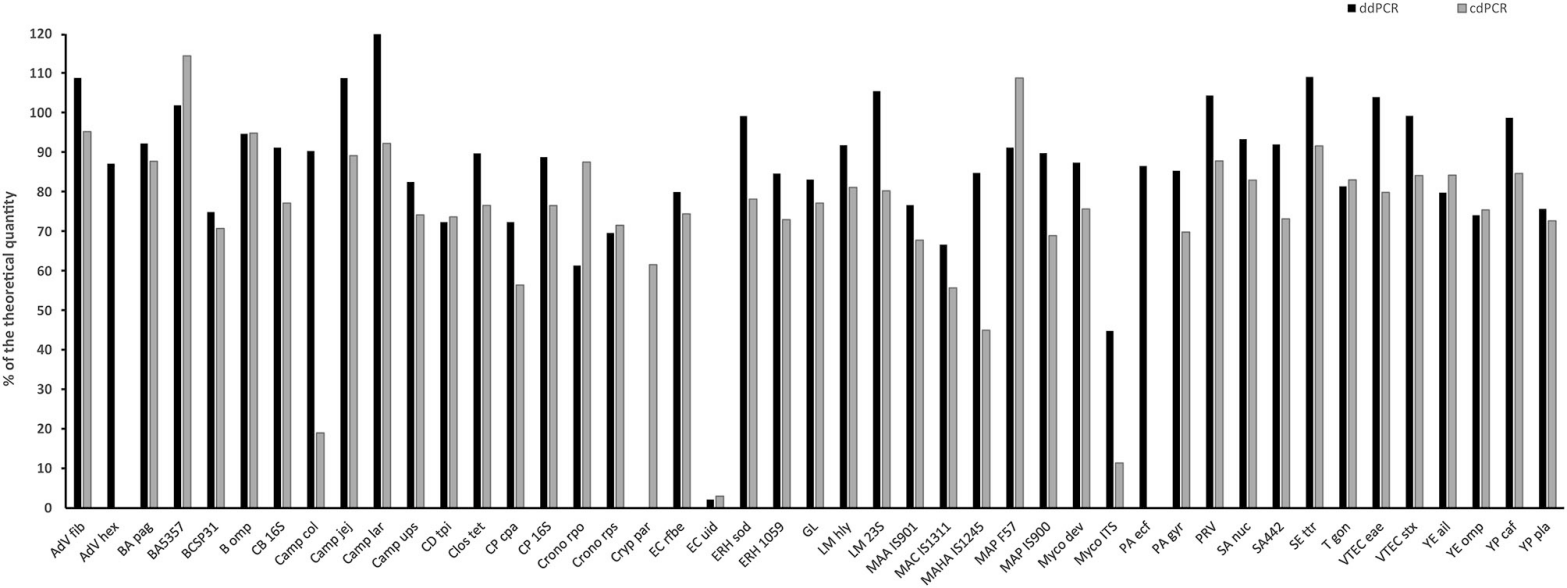
Comparison of quantity value of 45 linear plasmid standards using ddPCR and cdPCR. The bars represent the calculated MLE of plasmid standard concentration compared to the theoretical concentration (the values are given in percent).

Linear plasmid standard concentrations of 45 qPCR assays diluted based on spectrophotometric estimation in a range of 10^4^, 10^3^, and 10^2^ copies/μl were determined using ddPCR and cdPCR, MLEs of concentration were calculated and plotted against the theoretical quantity (Figure 3).

Percentage comparison of measured concentration values with the required concentrations, median, mean and confidence intervals of gained data indicated that the concentration values measured by both dPCR assays were mostly lower than the required concentrations, i.e., 100% (Tables 4, 5). The mean deviation from 100% were −14.06 percentage points for the ddPCR assay, and −25.81 percentage points for the cdPCR assay. The medians of both dPCR assays differed statistically significantly from 100% (*P* < 0.01; Wilcoxon one-sample test).

**Table 4:** Descriptive statistics regarding ddPCR and cdPCR assays.

**Statistics**	**ddPCR**	**cdPCR**	**Difference**
*N*	44	43	42
Minimum	2.17	2.98	−26.17
Maximum	120.36	114.45	71.43
Lower quartile (Q_1_)	77.52	70.78	2.24
Median	88.16	76.61	11.72
Upper quartile (Q_3_)	97.82	84.73	17.87
Quartile deviation (IQR)	20.30	13.95	15.63
Mean	85.94	74.19	11.41
Sample standard deviation (SD)	19.20	21.59	15.81
Standard error of the mean (SEM)	2.89	3.29	2.44
Lower limit of 95% confidence interval for the mean	80.10	67.54	6.48
Upper limit of 95% confidence interval for the mean	91.78	80.83	16.34

*Difference = ddPCR – cdPCR; IQR = Q_3_ – Q_1_*.

**Table 5:** Numbers of values lower than or greater than the theoretical quantity (100%).

**Criterion**	**ddPCR**	**cdPCR**
<100%	36 (81.82%)	41 (95.35%)
>100%	8 (18.18%)	2 (4.65%)

Regarding the difference, in which the concentrations of standards determined by cdPCR were subtracted from those determined by ddPCR, the mean of these values was 11.41 and the median was 11.72 (Table 4). The difference was positive for 32 samples (76.19%), suggesting that the values measured by ddPCR assay were mostly higher than those measured by the cdPCR assay, an average of 11.41 percentage points. The results of both dPCR assays on the same samples differed statistically significantly (*P* < 0.01; Wilcoxon paired test).

### Comparison of the Concentrations of Five Selected Linear Plasmid Standards Diluted Based on Spectrophotometric Estimation and Re-diluted Based on MLE Value Determined by ddPCR

**Figure 4:**
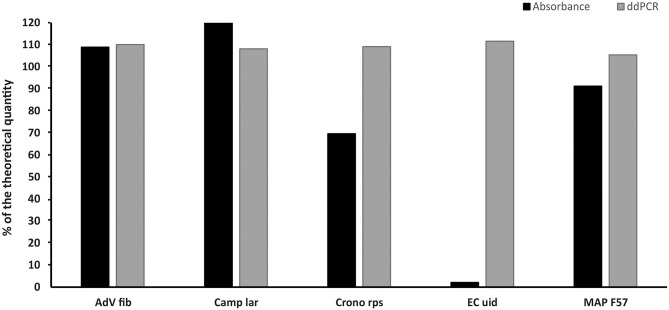
Comparison of the concentrations of selected standards diluted based on spectrophotometric estimation (Absorbance) and re-diluted based on value (MLE) determined by ddPCR (ddPCR). The bars represent the calculated MLE of plasmid standard concentration compared to the theoretical concentration (the values are given in percent).

The measured concentrations of selected plasmid standards (AdV Fib, Camp lar, Crono rps, EC uid, and MAP F57) diluted based on spectrophotometric estimation corresponded to a range of 2–120% of the theoretical quantity and showed a deviation of 9–98% from the theoretical quantity. After re-dilution based on value (MLE) measured by ddPCR, the concentrations of the standards differed from the theoretical quantity in the range of 5–12% (Figure 4).

### Evaluation of Stability of Linear Plasmid Standards During Long-Term Storage

**Figure 5:**
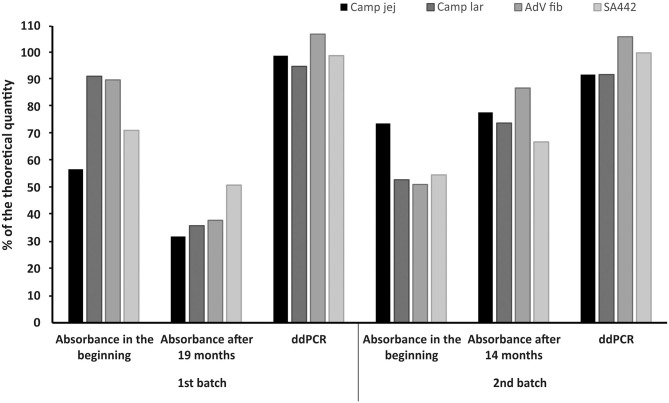
Comparison of the concentrations of linear plasmid standards diluted based on spectrophotometric estimation in the beginning (Absorbance in the beginning), 19 or 14 months thereafter (Absorbance after 19 or 14 months) and re-diluted based on value (MLE) determined by ddPCR (ddPCR). The bars represent the calculated MLE of plasmid standard concentration compared to the theoretical concentration (the values are given in percent).

The linear plasmid standards stored at −20°C for 19 months (first batch) showed a noticeable decrease in the concentration measured by ddPCR. In the case of storage for 14 months (second batch) at −20°C, changes in concentrations were also observed except for one standard whose concentration was not significantly altered. However, after recalibration (re-dilution) of these linear plasmid standards based on values from ddPCR, their concentrations again corresponded to a range of 92–107% of the theoretical quantity (Figure 5).

## Discussion

Nowadays, qPCR is a widely used method for the quantification of nucleic acids of various pathogenic microorganisms. For absolute quantification using qPCR, it is necessary to construct a calibration curve from accurately quantified quantification standards, from which the amount of the microbial pathogens in the sample is derived. However, currently, there is no established procedure for standard verification of these quantification standards. Laboratories quantifying microbial pathogens in the same samples by qPCR using quantification standards estimated by different methods may not obtain the same copy numbers (Kuypers and Jerome, 2017). Furthermore, the use of different quantification standards, which may be plasmid DNA constructs, synthetic RNA or DNA oligonucleotides spanning the complete PCR amplicon, cDNA cloned into plasmid, RNA or DNA from specific biological samples, international biological standards, etc. (Bustin et al., 2009), may also hinder the consistency of the results among various laboratories even when testing the same samples. Absolute quantification of qPCR quantification standards using dPCR that is not dependent on a calibration curve or highly efficient amplification (Streets and Huang, 2014), can provide improved accuracy and comparability of results when determining amounts of microbial pathogens using qPCR in various laboratories.

First, we examined the influence of conformational structure of the plasmid standard on its quantification. Since ddPCR did not allow accurate quantification of circular plasmid forms due to the so-called rain effect and thus creating an impossibility for setting the threshold correctly, we used qPCR for this purpose, where the linearized plasmids quantified by ddPCR were utilized for the calibration curve. Several studies already reported a difference in qPCR amplification depending on the conformation structure of the plasmid (Chen et al., 2007; Hou et al., 2010). In our study, the concentration estimation of the circular plasmid form reached in a range of about 20–30% of concentration of the linear plasmid form. These results indicate that the target sequence in the circular plasmid may not be sufficiently accessible for primers, probe and polymerase annealing probably due to the superhelical nature of the plasmid, which prevents full denaturation of the dsDNA and therefore a large portion of the template target sequence is not amplified. In order to accurately quantify the pathogens using plasmid standards, a linearized form is needed, since the circular form may hamper amplification of the target sequence.

The concentration estimates of the final quantification plasmid standards from dPCR analysis differed in the majority of cases from the concentrations expected based on spectrophotometric measurement of absorbance of the initial stock solution. The concentrations of the standards determined by dPCR were predominantly lower than the expected concentrations indicating an overestimation in spectrophotometric measurement of plasmid standard stock solutions. The results obtained suggest that these differences were probably associated with insufficient purity of the prepared stock solutions measured resulting in incorrect dilution of them to final quantification plasmid standards. Common contamination affecting spectrophotometric measurement may be chromosomal DNA, RNA, proteins, or salts (Sanders et al., 2011). Another explanation could be an inhomogeneity of the plasmid stock solution measured or the presence of plasmids without the target sequence insert, which contribute to higher apparent absorbance, however, this DNA does not carry any DNA of interest. Similar mismatches between expected concentrations of plasmid standards based on spectrophotometric estimates of stock solution and dPCR estimates were found in a recent study quantifying plasmid DNA standards used in qPCR for the detection of *Enterococcus* spp. (Sivaganesan et al., 2018). However, this deviation was attributed to manipulation of plasmid stock solution including restriction digestion, in addition to freezing and thawing of the materials for analysis. Another study also described disagreement between concentration estimates derived by spectrophotometry and dPCR (Sanders et al., 2011). In addition, differences in the concentration estimates between two batches of linear plasmid standards prepared by the same procedure were also observed.

Given the results suggesting an overestimation in spectrophotometric measurement of plasmid stock solution, we investigated various commercially available kits for plasmid DNA isolation to determine whether they are able to remove potential contamination. However, the results showed that the removal of contamination and thus attaining unaffected spectrophotometric measurement of standard stock solutions was not achieved after utilization of any tested isolation kits. Differences in concentration estimations of the individual standards were completely random considering using different isolation kits, as well as between concentration estimations of two standards tested using the same isolation kit. These data suggest that plasmid isolation kits from various manufacturers are generally not able to isolate solely plasmid DNA, but it is likely that some contaminating chromosomal DNA is also purified. Considering the molecular weight of plasmid and chromosomal DNA, even residual presence of chromosomal DNA can affect the final concentration estimation of a plasmid solution by absorbance measurement, which is then reflected in diluting the solution to the final quantification standards. Based on these findings, we recommend that absorbance measurement of standard stock solutions should be used only for a rough concentration estimate. Then, according to this estimate, the standard could be diluted to an order within the dynamic range of dPCR, which subsequently allows absolute quantification of the standard and its more accurate dilution.

Furthermore, we analyzed the linear plasmid standards of 45 qPCR assays using ddPCR and cdPCR platforms. The concentration estimations of the individual plasmid standards obtained by these two dPCR platforms differed significantly. The dynamic range of dPCR depends largely on the amount of the reaction partitions analyzed, and this amount is approximately 20,000 in both dPCR platforms (Pinheiro et al., 2012) utilized in this study, therefore, these two dPCR platforms should have approximately the same dynamic range. However, this was contradicted by the fact that in some cases in cdPCR software analysis of the two edge dilutions of the standard—expected concentration 10^4^ and 10^2^ copies/μl based on spectrophotometric measurement—the partition results were not clearly separated into negative and positive clusters indicating that these dilutions were already out of the dynamic range and resulting in deterioration of precision (Huggett et al., 2013). This led to a significant loss of linearity at these concentrations in cdPCR and therefore the calculation of MLE of standard concentrations from the three estimations may not give accurate results compared to ddPCR assay, whose response was linear over all three concentrations measured, and therefore probably provided more accurate results. As mentioned above, the absorbance measurements were overestimated in the vast majority of plasmid standard stock solutions, however, one value indicating the opposite was also recorded. In this experiment, the highest concentration estimation measured was recorded for a Camp lar standard with a value corresponding to 120% of the theoretical value when measured by ddPCR. The reason could be either an inhomogeneity of the plasmid stock solution in spectrophotometric measurement of absorbance or a slight underestimation in this measurement, which may also be caused by certain contaminants (Li et al., 2014). Nevertheless, according to this experiment, the approach of transformation of the optimized qPCR assays to dPCR platform seems to be robust and reliable. Only a few quantification standards failed to be analyzed using dPCR analysis—one standard using ddPCR platform and two standards using cdPCR platform out of 45 standards tested. Although, the PCR conditions optimized for qPCR (concentration of primers and probe) were set, it is possible that these conditions were not suitable for the dPCR assays. dPCR assays for which the conditions optimized for qPCR are not suitable, need to be further optimized, which involves, in addition to changes in primer and probe concentrations, changes of annealing temperature or ramp rate during amplification.

Since we concluded the calculation of MLE of concentration was more accurate in ddPCR than cdPCR, we used the values from ddPCR assays for re-dilution of plasmid standards, thus, to evaluate whether ddPCR is suitable for calibration of standards to the required quantity. Five standards were selected to include even those with extreme values, and after re-dilution, their concentration estimations again determined by ddPCR were approximately equal to the required concentration with a deviation of about 10%.

Freezing at −20°C is considered sufficient for storage of DNA for several months, for even longer storage period, freezing at −80°C is applied. To evaluate whether there are any changes in quantity of the linear plasmid standards during long-term storage at −20°C, stock solutions of the standards were again diluted based on the previously determined spectrophotometric estimations after 19 months for the first batch of the standards and after 14 months for the second batch. After 19 months of storage, there was a noticeable decrease in the concentration estimations compared to the standards measured in the beginning. After 14 months, the concentration estimate for one standard was not significantly changed, for the others there was even a slight increase in concentration estimations compared to the standards initially measured. Another study investigating the stability of DNA standards stored for 100 days at 4, 0, and −20°C using real-time PCR observed that freezing at −20°C provided the best storage conditions as it caused the least shift in the resulting *Ct* values (Roder et al., 2010). However, even such a shift would result in a significant deviation of the final sample concentrations if this DNA standard were used for a calibration curve in qPCR. In our study, after evaluating changes in standards concentration after long-term freezing, the standards were recalibrated (re-diluted) based on the values measured by the second ddPCR assay, and by a further additional ddPCR assay it was found that the concentration estimations of re-diluted standards corresponded to required concentrations with a deviation below 10%. Here we suggest that 10% variation (corresponding to 90–110% of the required quantity) in ddPCR results could be the criterion for the verified standard dilution. Long-term storage of standard is costs saving, but as can be seen, the quantity value of the standards may change over time when stored frozen. Therefore, the standards need to be checked by ddPCR at about 6 months whether they maintain the required quantity and recalibrate if necessary.

Results of this study showed that isolation of various plasmid DNAs, their quantification and storage in time represents a complex process, which can be biased on different levels even when maintaining standard operation procedures and standardized isolation kits. Variation may occur in different propagations of cells carrying plasmids, changes in stability of plasmids over time and proneness of the whole process to other factors, of which many of them are difficult to be anticipated. The biases in the concentration estimation of final quantification plasmid standards lead to heterogeneity of quantitative results within a single laboratory over time, which is reflected in determining the amount of a target sequence of microbial pathogens in samples derived from these qPCR standards. Therefore, an independent tool for the routine batch-to-batch control and verification of DNA standard quantity over time is needed. Here we suggest that ddPCR represents a suitable tool for such control. ddPCR is not affected by variation in the plasmid preparation process and can be universally used in any laboratory. It utilizes the same primers and probes as qPCR and amplifies only target DNA, without any interference from other DNA present in the sample. Therefore, standards of any qPCR method, in-house or commercial, can be verified by ddPCR without any laborious and time-consuming optimization. The accurate recalibration of quantification standards according to the ddPCR would result in obtaining more homogeneous, reproducible and comparable quantitative data by qPCR in various commercial and research laboratories over time. ddPCR could be even proposed as a reference method for the unification of DNA standards across different laboratories detecting identical pathogens via different methods. Quantities of DNA determined by these methods in different laboratories could therefore be more comparable and interpretation criteria would be unified. In other words, the interpretation of quantitative data from different laboratories would be presented in the “same language”.

## Data Availability Statement

All datasets generated for this study are included in the article/Supplementary Material.

## Author Contributions

MB performed the experiments, analysis, and interpretation of data and wrote the manuscript. VB performed the statistical and mathematical analysis. BB provided cdPCR equipment and helped to analyze cdPCR data. MBB provided part of ddPCR equipment and enabling work in her laboratory. PK designed the study was involved in data interpretation, provided biological material, and revised the manuscript. All authors contributed to the article and approved the submitted version.

## Conflict of Interest

The authors declare that the research was conducted in the absence of any commercial or financial relationships that could be construed as a potential conflict of interest.
