# Supplementary material for: Utilization of Digital PCR in Quantity Verification of Plasmid Standards Used in Quantitative PCR
Source: Front Mol Biosci. 2020 Jul 29;7:155. doi: 10.3389/fmolb.2020.00155 (PMC7403525; doi:10.3389/fmolb.2020.00155)
Supplement: Supplementary file 1 [file Table_1.DOCX]

**Supplementary Table 1. Primers and probes sequences of qPCR assays utilized in this study.**

| **Microorganism** | **Target locus** | **Abbreviation** | **Primer sequences 5´ to 3´ (forward; reverse)** | **Probe sequence** |
| --- | --- | --- | --- | --- |
| *Bacillus anthracis* | BA5357 | BA5357 | TCCAAGTTACAGTGTCGGCATATT;  CGGTGTAGGATTAAGTTTGGGCT | FAM – AGTTCATCATAAGCCAAGTCTGGGTAGTCAC – BHQ |
|  | pagA | BA pag | GAATTTGCGGTAACACTTCACTCC;  CGGATCAAGTATATGGGAATATAGCAAC | HEX – TCGAGCCTGTATCCACCCTCACTCTTCC – BHQ |
| *Brucella* spp. | BCSP31 | BCSP31 | TCGAGCTTGATGAGCTTG;  GACGACCTCATCTATAACATC | FAM – CTTGCGTGTATCCTCGTTCCA – BHQ |
|  | omp2 | B omp | ATTCCGATTCATCGATACCA;  TCGGGCGTAGATGGTAAATA | HEX – CGCAGACCACCGAGCTGGAT – BHQ |
| *Campylobacter coli* | glyA | Camp col | CATATTGTAAAACCAAAGCTTATCGTG;  AGTCCAGCAATGTGTGCAATG | HEX – TAAGCTCCAACTTCATCCGCAATCTCTCTAAATTT – BHQ |
| *Campylobacter jejuni* | hipO | Camp jej | TGCACCAGTGACTATGAATAACGA;  TCCAAAATCCTCACTTGCCATT | FAM – TTGCAACCTCACTAGCAAAATCCACAGCT – BHQ |
| *Campylobacter lari* | bipA | Camp lar | CATTTCAGCTTTTCTTTTGCCTAGT;  AAAACCGAACCATTTGAACACTTAG | FAM – ACCACACCAGTAAAATCATCAGGCACATCA – BHQ |
| *Campylobacter upsaliensis* | bipA | Camp ups | CTTAGATGTAGGAGATAGTGTCGTTTGTC;  CGGCAAAAACAATGCTTAAAGTT | HEX – TCCTCTCCCACTTGACCCACTTCACATT – BHQ |
| *Clostridium botulinum* | 16S rDNA | CB 16S | CTGACGACAACCATGCACCA;  GCGGAGCATGTGGTTTAATTCG | HEX – TGGACTTGACATCCCTTGCATAGCCTAGAGATAG – BHQ |
| *Clostridium difficile* | tpi | CD tpi | GGATTTAAAAGAAGCTACTAAGGGTA;  GGTGAAACTTCTCCTGTAAATGC | FAM – ATAGGTGCTCAAAATATGCACTTTGAAGA – BHQ |
| *Clostridium perfringens* | cpa | CP cpa | CTAGATATGAATGGCAAAGAGGAAACTA;  TTAGCAGGATGATATGGAGTATCTATATCTC | FAM – CAAGCTACATTCTATCTTGGAGAGGCTATGCACTAT – Cy5 |
| *Clostridium* spp. | 16S rDNA | CP 16S | CTGACGACAACCATGCACCA;  GCGGAGCATGTGGTTTAATTCG | HEX – AACGCGAAGAACCTTACCTACACTTGACATCC – BHQ |
| *Clostridium* *tetani* | tetox | Clos tet | GATACCTGGGGCATTATAACCTACT;  ATGAAATAGATTCTTTTGTTAGATCAGGTG | FAM – TTCCATCTTTCGGATAACCTACAATGTGCTCA-BHQ2 |
| *Cronobacter sakazakii* | rpsU gene 3' end and the primase (dnaG) gene 5' end | Crono rps | CGAGAATAAGCCGCGCATT;  GGGATATTGTCCCCTGAAACAG | BHQ – FAM – TCGGAAGCACGGCCTCTACA |
|  | rpoB | Crono rpo | TGATGCCGCAGGATATGAT;  GATCTCAGACAGCGGGTTGTT | BHQ – HEX – ACGCCAAGCCTATCTCCGCG |
| *Cryptosporidium* spp. | hsp70 | Cryp par | CAAGCTGCTATCTTAAATGG;  GAGCAACATCCAATAATAAGAG | FAM – TGAGCAATCCTCTGCCGTACAGG – BHQ |
| *Erysipelothrix rhusiopathiae* | ERH 1059 | ERH 1059 | CCCCATTCAAGTACGACGAC;  GATGGACGACTGAACCTAAAAC | FAM – CGATTGCGTCGGATGATACACCCTCT – BHQ |
|  | sodA | ERH sod | TCCGCACAGCAGTTCAAAAC;  CACCAGGCTCTTGACCTTTG | HEX – ACCATACGATGTTTTGGGAATTTCTATCCCCA – BHQ |
| *Escherichia coli* | uidA | EC uid | CAACGAACTGAACTGGCAGA;  CATTACGCTGCGATGGAT | BHQ – FAM – CCCGCCGGGAATGGTGATTAC |
|  | rfbE | EC rfbe | TTTCACACTTATTGGATGGTCTCAA;  CGATGAGTTTATCTGCAAGGTGAT | BHQ – HEX – AGGACCGCAGAGGAAAGAGAGGAATTAAGG |
| *Giardia lamblia* | β-giardin | GL | TCTATGTTCACCTCCACCCGTAC;  TTGCTGAGCTTGACCGCC | FAM – TCACCCAGACGATGGACAAGCCC – BHQ |
| *Human Adenovirus* | hexon | AdV hex | CCAGGACGCCTCGGAGTA;  AAACTTGTTATTCAGGCTGAAGTACGT | HEX – AGTTTGCCCGCGCCACCG – BHQ |
| *Human Adenovirus* serotype 40 and 41 | fiber | AdV fib | CGAACTTTCTCTCTTAATAGACGC;  GGCGTTCAATGGCTAGTGTAAG | FAM – TAATGCTGACACGGGCACTCTTCG – BHQ |
| *Listeria monocytogenes* | hlyIII | LM hly | CATGGCACCACCAGCATCT;  ATCCGCGTGTTTCTTTTCGA | HEX – CGCCTGCAAGTCCTAAGACGCCA – BHQ |
| *Listeria* spp. | 23S rDNA | LM 23S | AGGATAGGGAATCGCACGAA;  TTCGCGAGAAGCGGATTT | FAM – TCTCACACTCACTGCTTGGACGC – BHQ |
| *Mycobacterium avium* complex | IS1311 | MAC IS1311 | GCTGGACGCATTACGCAAT;  CGTCAGCTCCGCATCGAT | HEX – CCGCCGAGACGATTTATCAGGCAC – Cy5 |
| *Mycobacterium avium* subsp. *avium* | IS901 | MAA IS901 | GTGATCAAGCACCTTCGGAA;  GCTGCGAGTTGCTTGATGAG | HEX – AACAACATCGACACGATCGCCGACAA |
| *Mycobacterium avium* subsp. *hominissuis* and subsp. *avium* | IS1245 | MAHA IS1245 | CCGGATCTGCAAAGACCTC;  CGACACCACCCGATGATTC | FAM – CCGTTGGGTGATCAGCGCTTTC |
| *Mycobacterium avium* subsp. *paratuberculosis* | IS900 | MAP IS900 | GATGGCCGAAGGAGATTG;  CACAACCACCTCCGTAACC | FAM – ATTGGATCGCTGTGTAAGGACACGT |
|  | F57 | MAP F57 | GCCCATTTCATCGATACCC;  GTACCGAATGTTGTTGTCAC | FAM – CAATTCTCAGCTGCAACTCGAACACAC |
| *Mycobacterium* spp. | ITS | Myco ITS | CACTATTGGGCCCTGAGACAAC, TTGGGTCCTGAGGCAACAC, AGCAAGCCAGACACACTATTGG, TTGGGTCCTGAGGCAACAG;  GATGCTCGCAACCACTATCCA | FAM – TTGGTGGTGGGGTGTGGTGTTTGA – Cy5 |
| *Mycobacterium* *tuberculosis* | devR | Myco dev | CCGATGGCAACGGCATT;  GAGGATCAGACAGCGCAGATC | HEX – AACTGTGCCGCGATCTGTTGTCCC – BHQ |
| *Pseudomonas* *aeruginosa* | gyrB | PA gyr | GCAAGGAGGAGCTGTTCAAG;  GCTGGACGTTGAAGTGGAAT | FAM – CAACAAGACCGCGGTGAACG – BHQ |
|  | ecfX | PA ecf | CAGGATACTTTCGCCCAGTG;  GCGATCTGGAAAAGAAATGC | HEX – AATCGGTCGAGCAGCCGC – BHQ |
| *Pseudorabies virus* | gB | PRV | ACAAGTTCAAGGCCCACATCTAC;  GTCYGTGAAGCGGTTCGTGAT | FAM – ACGTCATCGTCACGACCGTGTGGTC – Cy5 |
| *Salmonella enterica* | ttr | SE ttr | CTCACCAGGAGATTACAACATGG;  AGCTCAGACCAAAAGTGACCATC | FAM – CACCGACGGCGAGACCGACTTT – BHQ1 |
| *Staphylococcus aureus* | SA442 | SA442 | CGACTAAATAAACGCTCATTCG;  CTCTCGTATGACCAGCTTCG | FAM – CGTTGCATCGGAAACATTGTGTTC – BHQ |
|  | nuc | SA nuc | CAGCAAATGCATCACAAACAG;  GCTTTAATTAATGTCGCAGGTTC | HEX – CGTAAATAGAAGTGGTTCTGAAGATCC – BHQ |
| *Toxoplasma gondii* | B1 gene | T gon | TCGAAGCTGAGATGCTCAAAGTC;  AATCCACGTCTGGGAAGAACTC | FAM – ACCGCGAGATGCACCCGCA – BHQ |
| Verotoxigenic *Escherichia coli* | stx 1, 2 | VTEC stx | TTTGTYACTGTSACAGCWGAAGCYTTACG;  CCCCAGTTCARWGTRAGRTCMACRTC^1^ | FAM – CTGGATGATCTCAGTGGGCGTTCTTATGTAA – BHQ,  FAM – TCGTCAGGCACTGTCTGAAACTGCTCC – BHQ |
|  | eae | VTEC eae | CATTGATCAGGATTTTTCTGGTGATA;  CTCATGCGGAAATAGCCGTTA | HEX – ATACTCTCGCCAGTATTCGCCACCAATACC – BHQ |
| *Yersinia* *enterocolitica* | ail | YE ail | ATGATAACTGGGGAGTAATAGGTTCG;  CCCAGTAATCCATAAAGGCTAACATAT | HEX – TCTATGGCAGTAATAAGTTTGGTCACGGTGATCT – BHQ |
| *Yersinia* *pestis* | caf1 | YP caf | AACCAGCCCGCATCACTCTTA;  TTCCTGTTTTATAGCCGCCAAGA | FAM – AGGCGCTCCAATTACAATTATGGACAATGGA – BHQ |
|  | pla | YP pla | TAAATTCAGCGACTGGGTTCGG;  AAAGACTTTGGCATTAGGTGTGAC | HEX – AGAAGACATCCGGCTCACGTTATTATGGTA – BHQ |
| *Yersinia* spp*.* | ompF | YE omp | AGRCCAGTAGAACGRCCAG;  GYCTGGGCTTYGCKGGTCTG | FAM – TTGTCAGAGTTGGAGATTGAATCACCACCGA – BHQ |

^1^ For degenerate sequences, Y is (C, T), S is (C, G), W is (A, T), R is (A, G) and M is (A, C).
